# Elevated metallothionein expression in long-lived species

**DOI:** 10.18632/aging.203831

**Published:** 2022-01-13

**Authors:** Marco Malavolta, Kamil Pabis

**Affiliations:** 1 Advanced Technology Center for Aging Research and Geriatric Mouse Clinic, IRCCS INRCA, Ancona 60121, Italy; 2 Max Planck Institute for Biophysical Chemistry, Göttingen, 37077, Germany

**Keywords:** metallothioneins, longevity, geroscience, mammals, long-lived species

Metallothioneins (MTs) are a family of small (6-7 kDa) cysteine-rich metal-binding proteins that have been extensively studied for their function in homeostatic regulation of zinc (Zn) and copper (Cu) as well as for their role in heavy metal detoxification.

Further studies have contributed to move the attention around the function of MTs from toxicology to biogerontology [1]. Indeed, MTs have been shown to protect cells against oxidative, UV and even viral [2] forms of damage. The gene expression of MTs increases in multiple tissues in response to aging and to anti-aging interventions including caloric restriction (CR) and inhibition of the insulin/insulin-like signaling (IIS) pathway [1].

MTs display all the features of strong candidate longevity genes. Overexpression of MTs was found to lengthen mouse lifespan [3] while MT-knockout shortens lifespan and promotes cancer [4]. The lifespan extension mediated by overexpression of MTs has been further observed even in a mouse model of amyotrophic lateral sclerosis (Tokuda E, et al. Mol Genet 2014) and after cardiac-specific overexpression of the human MT2 isoform in mice [5].

The potential mechanisms underlying these beneficial effects of MTs are still not completely understood. Protection from multiple stressors by MTs is in line with modern stress resistance and damage-based theories of aging. For example, we know that fibroblasts derived from long-lived species resist cell death induced by Cd, paraquat or other stressors and that long-lived mice show higher expression of cytoprotective genes regulated by the Nrf2 transcription factor. MTs are among the genes induced by Nrf2, placing them as effectors of this central geroprotective transcriptional network. Taking into account that loss of metallostasis and accumulation of cadmium (Cd) in multiple tissues are common features of aged organisms, it could be also argued that MTs protect from age-related Cd toxicity and metal dyshomeostasis.

In a recent paper we further strengthened the role of MTs in aging addressing the interaction among MTs, Cd and longevity [6]. We found that long-lived species accumulated Cd faster than expected by chronologic age, implying an additional factor was at play. An extensive meta-analysis and assembly of novel datasets provided evidence that long-lived mammals express more MTs at the protein and mRNA level than short-lived ones, which may explain, at least in part, the “paradoxical” faster accumulation of Cd. To further support this hypothesis, we measured Cd in several long-lived mouse models with elevated MT expression. Most of them were found to have elevated hepatic and renal Cd content with the strongest results in MT-transgenic mice. These results suggest that long-lived species have evolved a more efficient induction of MTs in response to damage, which in turn contributes to faster accumulation of tissue Cd levels. Since Cd is tightly bound to MTs, the toxicity of the metal is suppressed and its longevity costs are likely offset by the beneficial effects of MTs. It could be additionally argued that the protective effect of MTs involves also Cu, which is an essential trace element known to accumulate with aging and able to induce premature senescence in human fibroblasts (Matos L, et al. Age (Dordr) 2012).

In spite of this considerable knowledge on the role played by MTs in longevity, modulation of their expression and testing in aged non-transgenic animals is still elusive, and this is a critical step toward utilizing these mechanisms for the treatment of age-related conditions in humans.

In worm models of neurodegenerative disorders indirect induction of MTs by Zn, progesterone, quercetin, dexamethasone and apomorphine reduced the burden associated with Amyloid β and α-synuclein while knockdown of MTs resulted in a partial loss of bioactivity of these compounds [7]. However, these and most other known MT-inducing compounds are also non-specific inducers of Nrf2 expression or are known to interfere with many other pathways thus complicating their therapeutic translation based on the specific target. By contrast, direct induction of MTs by gene therapy has been limited only to chemically-induced liver fibrosis mouse models with interesting positive results (Jiang Y and Kang YJ, Mol Ther 2004). Indeed, the therapy reversed the fibrosis along with increased hepatocyte regeneration in few days.

This technology, or alternative safe gene-delivery systems, to overexpress MTs “in vivo” clearly poses some challenges. Given the complexity of the aging process, their effectiveness may produce unsatisfactory results or unexpected side-effects. However, methods and protocols are now finally available to implement studies in geriatric mice or other preclinical models of aging. It has been recently shown that adeno-associated virus (AAV)-based therapies employing longevity associated genes are safe and can been successfully applied in the simultaneous treatment of several age-related diseases [8].

Previous studies on MTs have seen a shift from toxicology to biogerontology, we expect that the next step will be a shift from biogerontology to geroscience, which aims to directly extend organismal healthspan and ameliorate the burden of age-related diseases.
